# Comparison of virtual and true ECG-gated coronary CT angiography in patients with acute chest pain: a prospective cohort study

**DOI:** 10.3389/fradi.2026.1893538

**Published:** 2026-10-07

**Authors:** Wenyan Wei, Lin Lu, Yan Wu, Qianye Fang, Yuankun Li, Junyue Zhou, Runmeng Li, Dan Han, Tengfei Ke, Wen Zhao

**Affiliations:** 1 Department of Medical lmaging, The First Affiliated Hospital of Kunming Medical University, Kunming, China; 2 Department of Radiology, Yunnan Cancer Hospital (The Third Affiliated Hospital of Kunming Medical University), Kunming, China

**Keywords:** chest pain, computed tomography angiography, coronary angiography, electrocardiography, image quality, radiation dose

## Abstract

**Purpose:**

To compare the clinical value of true electrocardiogram-gated and virtual electrocardiogram-gated coronary computed tomography angiography (CCTA), and to provide a reasonable and effective imaging strategy for patients with acute chest pain.

**Methods:**

A total of 254 patients with acute chest pain and suspected coronary artery disease who underwent CCTA were prospectively enrolled. Patients were alternately assigned according to examination date to the virtual ECG-gated group (*n* = 126), or the true ECG-gated group (*n* = 128). Examination time and radiation dose were recorded. Objectiveand subjective image quality parameters were evaluated. Objective parameters included image background noise, CT values of the aorta and coronary artery branches, signal-to-noise ratio (SNR), and contrast-to-noise ratio (CNR). Subjective image quality was assessed using a 5-point scale. In patients who underwent invasive coronary angiography (ICA) within 30 days after CCTA, diagnostic agreement was evaluated using ICA as the reference standard. Diagnostic performance was assessed at the vessel level using receiver operating characteristic (ROC) curve analysis, with the four major coronary arteries from each patient considered as the units of analysis.

**Results:**

(1) The virtual ECG-gated group demonstrated significantly improved examination efficiency compared with the true ECG-gated group, with a shorter total examination time (2.83 ± 0.37 min vs. 4.15 ± 0.49 min, *P* < 0.001) and positioning time (0.89 ± 0.26 min vs. 2.03 ± 0.35 min, *P* < 0.001). However, the radiation dose was higher in the virtual ECG-gated group than in the true ECG-gated group (3.58 ± 0.69 mSv vs. 3.18 ± 0.54 mSv, *P* < 0.001). (2) Image quality analysis showed that the virtual ECG-gated group had lower image noise than the true ECG-gated group, as well as higher SNR and CNR values for the aorta and coronary artery branches (all *P* < 0.001). However, CT values and subjective image quality scores were comparable between the two groups (all *P* > 0.05). (3) In the ICA-validated subgroup, the virtual ECG-gated group showed a higher diagnostic agreement rate than the true ECG-gated group (92.00% vs. 85.58%), with corresponding vessel-level AUC values of 0.89 and 0.82, respectively. However, the virtual ECG-gated group was associated with a higher radiation dose than the true ECG-gated group (3.58 ± 0.69 mSv vs. 3.18 ± 0.54 mSv), representing a trade-off for achieving an ECG-independent acquisition strategy and maintaining diagnostic image quality.

**Conclusion:**

Virtual ECG-gated CCTA can significantly improve examination efficiency while maintaining comparable image quality and diagnostic performance to true ECG-gated CCTA. Although this approach was associated with a modest increase in radiation dose, this represents a trade-off for reducing dependence on real-time ECG acquisition and simplifying the CCTA workflow. Therefore, virtual ECG-gated CCTA may serve as a feasible and efficient imaging strategy for selected patients with acute chest pain, particularly in clinical scenarios requiring rapid evaluation or simplified workflow.

## 1 Introduction

Coronary artery disease (CAD) is one of the most common forms of atherosclerotic cardiovascular disease, with increasing prevalence and mortality worldwide. It remains one of the leading causes of death globally (1). Acute chest pain is a common reason for presentation to the emergency department, accounting for approximately 4.7% of all emergency department visits in the United States (2). Among the underlying causes, acute coronary syndrome (ACS) represents one of the most common and clinically critical conditions (3). In current clinical practice, patients presenting with acute chest pain are initially evaluated using electrocardiography and high-sensitivity cardiac troponin (hs-cTn), while noninvasive imaging is often performed for further assessment of myocardial ischemia (4). In recent years, both the European Society of Cardiology (ESC) and the American Heart Association (AHA) guidelines (3, 5) have recommended CCTA as an important noninvasive imaging modality for the evaluation of patients with ACS.

CCTA enables accurate visualization of the entire coronary artery tree, quantitative assessment of coronary artery stenosis, and characterization of atherosclerotic plaque composition, thereby providing valuable information for precise diagnosis, risk stratification, and prognostic evaluation (6). With the advantages of noninvasiveness and high spatial resolution, CCTA has been widely established as a first-line imaging modality for the diagnosis, risk assessment, and treatment planning of coronary artery disease (7). With rapid advances in CT technology, the feasibility of conventional CCTA has been substantially improved, although appropriate ECG synchronization remains essential for reliable coronary assessment. Nevertheless, CCTA acquisition remains highly dependent on electrocardiogram-gated (ECG-gated) techniques (8, 9).

In ECG-gated CCTA, electrocardiographic electrodes must be placed on the patient's body surface before scanning to enable real-time ECG signal acquisition, and image data are then acquired at specific phases of the cardiac cycle. These additional steps increase procedural complexity and prolong examination time (10). Moreover, the placement of surface ECG electrodes may require exposure of private body areas, particularly the breasts in female patients, which can increase patient anxiety and discomfort. For patients with acute chest pain, CCTA also faces two important practical challenges. First, examination time should be minimized to facilitate rapid clinical decision-making. In emergency settings, reducing non-diagnostic procedural steps and shortening imaging workflow may help accelerate the evaluation process, allowing earlier risk stratification and treatment decisions for patients with suspected coronary artery disease. Although the absolute time reduction may appear modest in routine practice, improvements in workflow efficiency may be particularly valuable in time-sensitive clinical scenarios where multiple diagnostic procedures and therapeutic decisions need to be coordinated. Second, reliable ECG signal acquisition may be difficult in certain special situations, including severe chest pain with poor patient cooperation, extensive chest burns or scars, cardiac tamponade, or ongoing life-support measures. To address these workflow-related limitations, an ECG-less CCTA technique using virtual ECG gating has been developed and explored in clinical practice. By eliminating the need for real-time ECG signal acquisition, this technique may simplify the workflow, shorten examination time, and expand the applicability of CCTA in acute and complex clinical settings (11).

Previous studies have demonstrated that ECG-independent CCTA using wide-detector and single-source CT systems is feasible; however, these approaches were mainly validated in patients with heart rates below 75 beats per minute (12, 13). The introduction of 16-cm wide-detector CT systems enables whole-heart coverage within a single cardiac cycle and increases the likelihood of successful virtual ECG-gated CCTA across patients with varying heart rates, thereby expanding its potential clinical applicability. Intelligent reconstruction algorithms provides a novel approach for acquiring diagnostically acceptable coronary images without conventional ECG signal acquisition (14). Previous studies comparing virtual ECG-gated CCTA with conventional true ECG-gated CCTA have suggested its feasibility in maintaining image quality and improving examination efficiency (15). However, systematic comparisons between virtual ECG-gated CCTA and conventional true ECG-gated CCTA in patients with acute chest pain remain limited, particularly regarding examination efficiency, image quality, radiation exposure, and diagnostic performance.

Therefore, this prospective controlled study aimed to comprehensively evaluate whether virtual ECG-gated CCTA could provide comparable diagnostic performance to conventional true ECG-gated CCTA while improving examination efficiency in patients with acute chest pain. Multiple aspects, including examination workflow, radiation exposure, image quality, and diagnostic performance, were assessed. The findings may provide evidence for the clinical applicability and protocol optimization of virtual ECG-gated CCTA in time-sensitive clinical settings. The overview of this study is shown in Figure 1.

**Figure 1:**
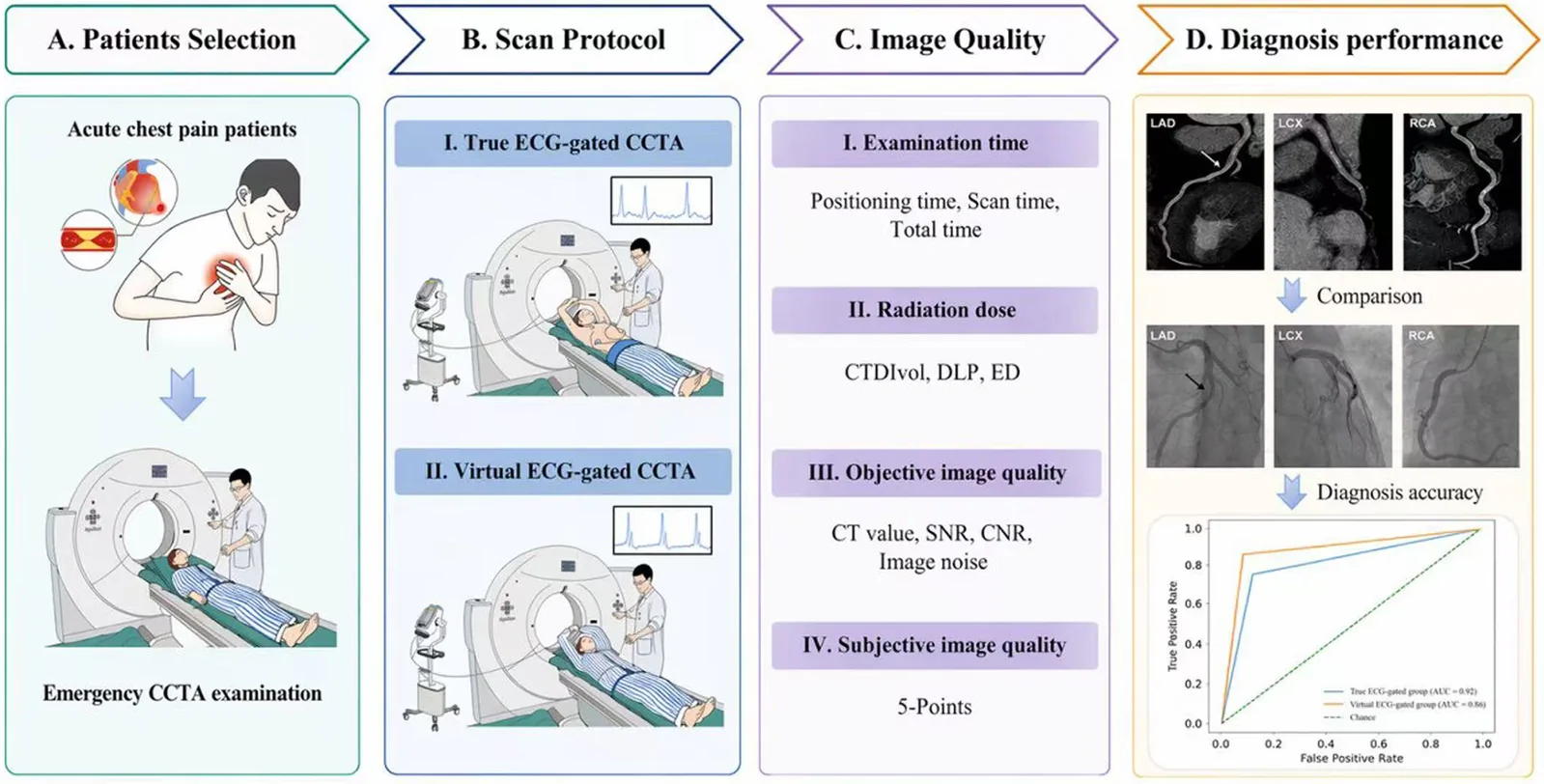
Overview of the study.

## 2 Materials and methods

### 2.1 Patients

This prospective cohort study was approved by our Institutional Review Board (K2025-L-155) and was conducted in accordance with the Declaration of Helsinki. Written informed consent was obtained from all participants. Patients with acute chest pain and suspected CAD who were referred to the Department of Medical Imaging for CCTA between February 2, 2026, and April 10, 2026, were prospectively enrolled. Exclusion criteria: (1) age ≤ 18 years; (2) allergy to iodinated contrast media; (3) prior coronary revascularization; (4) severe arrhythmia; (5) severe renal insufficiency.

Patients were prospectively assigned to the virtual ECG-gated or true ECG-gated CCTA groups according to a predefined alternating schedule based on examination date. Finally, a total of 254 patients were included in this study, comprising 126 patients in the virtual ECG-gated group and 128 patients in the true ECG-gated group. The patient flowchart of this study is shown in Figure 2.

**Figure 2:**
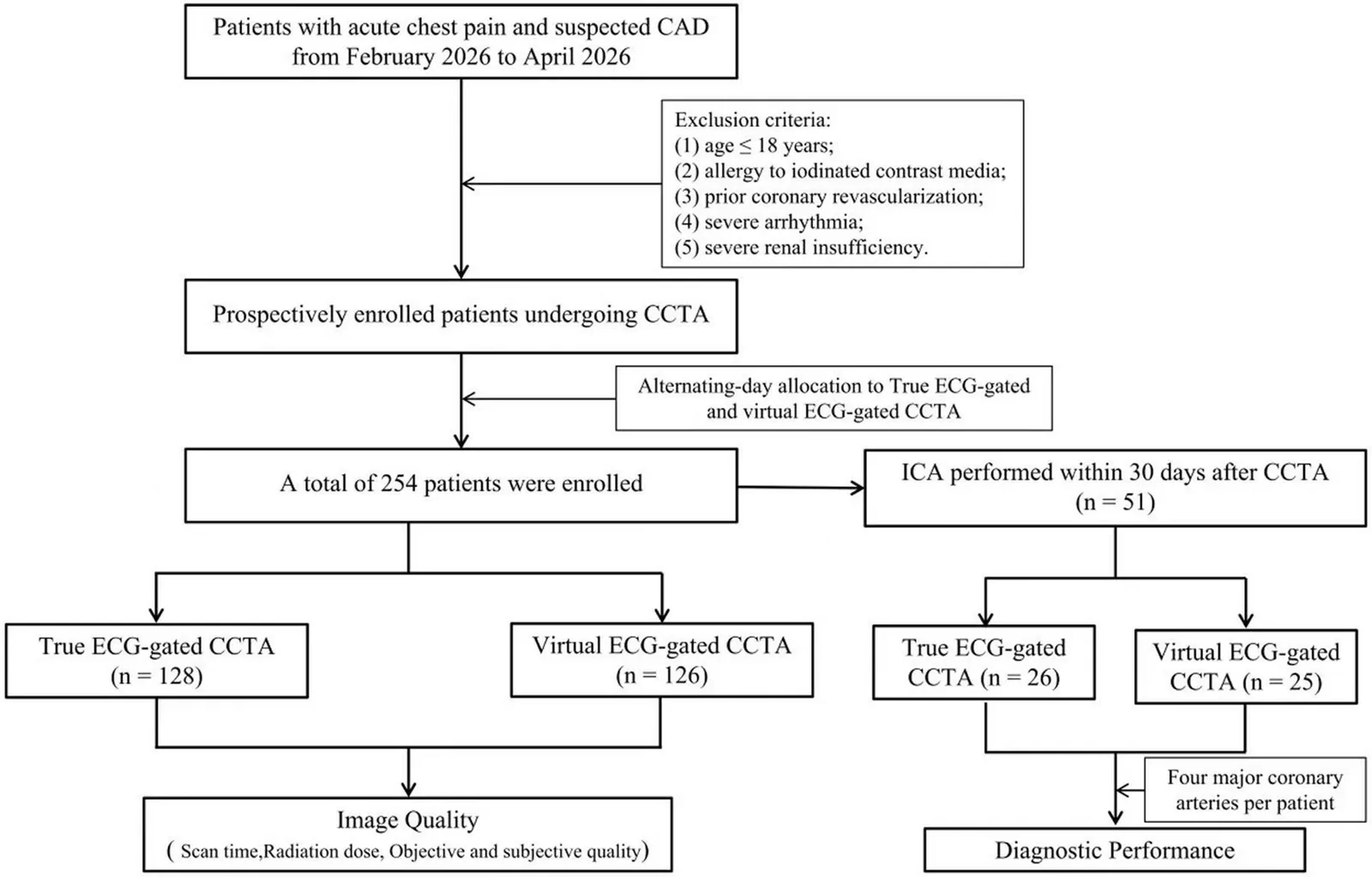
Patients flowchart of this study.

### 2.2 CCTA acquisition protocol

No patients received β-blockers or nitroglycerin before CCTA examination. Before scanning, a 20-gauge intravenous catheter was placed. Patients were positioned supine and instructed to raise both arms above the head, with the arm containing the intravenous catheter kept straight and not flexed. For patients who were unable to fully raise both arms, the arms were positioned alongside the body.

All CCTA examinations were performed using a prospective scanning mode. The scan length was selected according to cardiac size and was set at 12, 14, or 16 cm. Iohexol injection (Omnipaque, 350 mg I/mL, GE Healthcare, China), with an iodine concentration of 350 mg/mL, was administered through the median cubital vein at a flow rate of 4–6 mL/s, followed by 40 mL of normal saline at the same flow rate. The contrast medium volume and injection flow rate were adjusted according to the patient's body mass index (BMI) and the condition of the intended venous access. Bolus tracking was used, and CCTA acquisition was initiated 7.0 s after the CT attenuation of the aortic root exceeded 120 HU. All patients underwent CCTA using a 256-row CT scanner (Revolution CT, GE Medical Systems, WI, USA). In both groups, tube voltage was automatically selected according to BMI, with settings of 80, 100, or 120 kV, and automatic tube current modulation was applied.

In the virtual ECG-gated group, ECG-less CCTA was performed without placement of ECG electrodes on the patient's body surface. Before scanning, the patient's heart rate was measured using a finger pulse oximetry, and and a simulated heart rate slightly lower than the measured value was entered into the virtual ECG settings. A regular virtual ECG signal with no beat-to-beat heart rate variability was then generated. This virtual ECG did not represent the patient's actual ECG waveform. Only its cardiac cycle length was related to the heart rate measured by the finger pulse oximetry, and the R - R intervals of the virtual and true ECG signals were not temporally aligned. The virtual ECG was used solely to provide the scan-triggering conditions. In the virtual ECG-triggered group, CT data were acquired at one complete cardiac cycle, corresponding to 0% - 100% of the virtual R - R interval (16). Specifically, data acquisition commenced at the 0% phase of the virtual R–R interval and ended at 100%. Notably, the 0% and 100% phases did not necessarily correspond to fixed or equivalent physiologic cardiac phases across patients. The simulated heart rate used to generate the virtual ECG for scan triggering was intentionally set below the patient's measured pre-scan heart rate. This approach was adopted to account for potential changes in heart rate between the pre-scan measurement and image acquisition, as well as for the unknown rhythm status during scanning, thereby ensuring that the acquired data included at least one complete cardiac cycle for subsequent processing and generation of diagnostically acceptable CCTA images (16).

Data were acquired using the one-beat best-phase scanning mode, with the noise index (NI) set to 18 HU. In the true ECG-gated group, the data acquisition window was automatically gated according to the R–R interval determined by the patient's heart rate. In the true ECG-gated group, data were acquired during the optimal diastolic phase (70%–80% of the R–R interval) and optimal systolic phase (30%–60% of the R–R interval). The NI was set at 20 HU, and all other scanning parameters were kept consistent with those in the virtual ECG-gated group.

Among all cardiac phases, the image with the least motion artifact was automatically selected using SmartPhase technology (GE Healthcare), based on image-based motion-artifact assessment rather than cardiac-phase selection. The image selected by Smart Phase was subsequently processed using the SnapShot Freeze (SSF) coronary motion-correction algorithm (GE Healthcare), which integrates data from adjacent cardiac phases, regardless of whether they belong to the same cardiac cycle, to correct and compensate for coronary artery motion and thereby improve image quality. This technique was mainly applied to vessel segments with apparent motion artifacts. All images were reconstructed using SSF to minimize motion artifacts at the selected phase, as shown in Figure 1B.

### 2.3 Image quality

#### 2.3.1 Examination time assessment

Examination time was recorded by the CT technologists responsible for patient positioning and scanning. The technologists recorded all relevant time points according to a standardized workflow and were not involved in subsequent image analysis or statistical evaluation. Patient positioning and scanning procedures were performed according to the institutional routine workflow. All examinations were performed in the same CT room, and all participating technologists had more than 5 years of experience in CT operation. The routine workflow of the scanning room remained stable throughout the study period.

The examination time was recorded for each patient and divided into the following three intervals: (1) Positioning time was defined as the interval from the patient lying on the CT examination table to completion of patient positioning by the CT technologist. This interval represented the preparation time before formal scanning. (2) Scan time was defined as the interval from the start of scanning to completion of raw data acquisition, excluding image post-processing and three-dimensional reconstruction. (3) Total examination time was defined as the interval from the patient lying on the CT examination table to completion of image acquisition, and was calculated as the sum of positioning time and scan time.

#### 2.3.2 Radiation dose assessment

Radiation dose parameters were recorded for each CCTA examination, including dose-length product (DLP), volume CT dose index (CTDIvol), and effective dose (ED). The ED was calculated using the following formula: ED = DLP×k, where k represents the conversion coefficient for chest CT examinations, with a value of 0.014 mSv·mGy⁻^1^·cm⁻^1^ (14).

#### 2.3.3 Objective image quality

Objective image quality was assessed independently by two radiologists with 5 and 12 years of experience in cardiovascular imaging, respectively, who were blinded to the scanning protocol and clinical information. In cases of substantial disagreement, measurements were reviewed and adjudicated by a senior radiologist with 20 years of diagnostic experience. On axial CCTA images, CT values and standard deviations (SD) were measured at the following sites: the aortic root (AO), left main coronary artery (LM), mid-segment of the left anterior descending artery (LAD-M), distal segment of the left anterior descending artery (LAD-D), mid-segment of the left circumflex artery (LCX-M), distal segment of the left circumflex artery (LCX-D), proximal segment of the right coronary artery (RCA-P), mid-segment of the right coronary artery (RCA-M), distal segment of the right coronary artery (RCA-D), and the left pectoralis major muscle. For vascular measurements, regions of interest were placed within the vessel lumen, with an area of 20–400 mm^2^, covering more than half of the luminal area where possible, while carefully avoiding the vessel wall and atherosclerotic plaques. The standard deviation of the ROI placed in the pectoralis major muscle was used as the image noise (17). The mean CT values and image noise obtained from the two radiologists were used for further analysis. SNR and CNR were calculated for each coronary artery segment as follows (18):

SNR=CTvalueofROI/imagenoise

CNR=CTvalueofROI−(CTvalueofpectoralismajormuscle)/imagenoise

#### 2.3.4 Subjective image quality

Subjective image quality was independently evaluated by the same two radiologists in a double-blinded manner. In cases of disagreement, the final score was determined by a third senior radiologist. Image quality was assessed using a 5-point scale as follows (19): 5–excellent image quality, with sharply defined coronary artery margins and no obvious artifacts; 4–good image quality, with clearly visible coronary artery margins and mild artifacts that did not affect diagnosis; 3–moderate image quality, with acceptable visualization of the coronary artery margins and artifacts present but still allowing diagnostic evaluation; 2–poor image quality, with blurred coronary artery margins and obvious artifacts that affected diagnosis; 1–very poor image quality, with unclear visualization of the coronary arteries and images considered non-diagnostic. The evaluated vessels included the main coronary arteries and their major branches: LM, LAD, first diagonal branch (D1), LCX, first obtuse marginal branch (OM1), RCA, posterior descending artery (PDA), posterolateral branch (PLB), and ramus intermedius (RI). The ramus intermedius was absent in some patients.

#### 2.3.5 Diagnostic performance

Invasive coronary angiography (ICA) was performed when clinically indicated at the discretion of the treating cardiologist, in accordance with contemporary guideline recommendations (20), based on an integrated assessment of the patient's clinical presentation, electrocardiographic findings, cardiac troponin levels, and CCTA findings. Patients with suspected obstructive CAD for whom invasive confirmation was considered necessary to establish the diagnosis and/or guide subsequent therapeutic management underwent ICA.

CCTA images were interpreted by a thoracic radiologist with 12 years of experience, who was blinded to patients’ clinical information and ICA results. Significant coronary artery stenosis was defined as luminal diameter reduction of ≥50% according to previous studies (21). For patients who underwent ICA within 30 days after CCTA, ICA findings were used as the reference standard for evaluating the diagnostic performance of CCTA. Diagnostic assessment was performed on a per-vessel basis involving the four major coronary arteries: LM, LAD, LCX, and RCA. Because multiple coronary vessels were obtained from the same patient and were therefore not statistically independent, vessel-level diagnostic analyses were performed while accounting for intra-patient clustering. Diagnostic performance metrics were evaluated using patient-level cluster bootstrap resampling, and mixed-effects logistic regression models incorporating patient ID as a random effect were used to account for correlations among coronary arteries within the same patient.

#### 2.3.6 Statistical analysis

All statistical analyses were performed using R software (version 4.3.1) and SPSS software (version 26.0). Continuous variables were expressed as mean ± standard deviation (SD), and categorical variables were presented as frequencies and percentages. Comparisons of continuous variables between the virtual ECG-gated and true ECG-gated groups were performed using the independent-samples t test. Categorical variables were compared using the chi-square test or Fisher's exact test, as appropriate. Interobserver agreement for objective quantitative measurements was evaluated using the intraclass correlation coefficient (ICC). ICC values were interpreted as follows: <0.50 indicated poor agreement, 0.50–0.75 moderate agreement, 0.75–0.90 good agreement, and >0.90 excellent agreement. For subjective image quality evaluation, scores based on a 5-point ordinal scale were compared between groups using the Mann–Whitney U test. Interobserver agreement for subjective image quality scores was assessed using weighted kappa statistics. Weighted kappa values were interpreted as follows: <0.20 indicated slight agreement, 0.21–0.40 fair agreement, 0.41–0.60 moderate agreement, 0.61–0.80 substantial agreement, and >0.80 almost perfect agreement. All statistical tests were two-sided, and a *P* value <0.05 was considered statistically significant.

For diagnostic performance analysis, coronary arteries were considered the unit of analysis, and four major coronary arteries from each patient were evaluated, including the LM, LAD, LCX, and RCA. To account for the correlation among multiple vessels within the same patient, patient-level cluster bootstrap resampling with 1000 iterations was performed to estimate 95% confidence intervals for diagnostic performance measures, including sensitivity, specificity, and area under the receiver operating characteristic curve (AUC). Additionally, mixed-effects logistic regression models were constructed with CCTA diagnosis as the predictor variable and ICA findings as the outcome variable, while including patient ID as a random intercept to account for within-patient clustering effects. Receiver operating characteristic (ROC) curves were generated based on vessel-level diagnostic classification. The AUC values for virtual ECG-gated and true ECG-gated CCTA were reported with 95% confidence intervals derived from cluster bootstrap resampling.

## 3 Results

### 3.2 Patient characteristics

A total of 254 patients were enrolled in this study, including 126 patients in the virtual ECG-gated group and 128 patients in the true ECG-gated group. There were no significant differences in age, sex, smoking status, hypertension, diabetes mellitus, height, body weight, BMI, or heart rate between the virtual ECG-gated and true ECG-gated groups (Table 1).

**Table 1:** Comparison of clinical characteristics and iodine contrast media strategies between two groups.

Clinical variables	Virtual ECG -gated group (*n* = 126)	True ECG –gated group (*n* = 128)	*P*-value
Age (years)	59.91 ± 11.80	60.63 ± 14.10	0.656
Sex (n, %)			0.456
Male	74 (58.7%)	81 (63.3%)	
Female	52 (41.3%)	47 (36.7%)	
Smoking (n, %)			0.239
Yes	48 (38.1%)	58 (45.3%)	
No	78 (61.9%)	70 (54.7%)	
Hypertension (n, %)			0.318
Yes	66 (52.4%)	59 (46.1%)	
No	60 (47.6%)	69 (53.9%)	
Diabetes (n, %)			0.630
Yes	34 (27.0%)	38 (29.7%)	
No	92 (73.0%)	90 (70.3%)	
Height (m)	1.62 ± 0.06	1.63 ± 0.09	0.262
Weight (kg)	64.55 ± 6.32	65.02 ± 11.80	0.691
BMI (kg/m^2^)	24.57 ± 1.69	24.34 ± 3.27	0.481
Heart rate (bpm)	79.20 ± 10.41	78.29 ± 10.04	0.479
Injection flow rate (mL/s)	4.80 ± 0.29	4.78 ± 0.31	0.601
Volume of ICM (mL)	62.32 ± 6.48	62.73 ± 10.83	0.715

ECG: Electrocardiogram; BMI: Body Mass Index; ICM: Iodine Contrast Media.

Continuous variables are presented as mean ± standard deviation.

Categorical variables are presented as *n* (%).

### 3.2 Examination time and radiation dose

There were no significant differences in contrast injection rate or contrast volume between the virtual ECG-gated and true ECG-gated groups (Table 1). The virtual ECG-gated group showed significantly shorter total examination time and positioning time than the true ECG-gated group, whereas scan time did not differ significantly between the two groups (Table 2). The DLP, CTDIvol, and effective dose were all significantly higher in the virtual ECG-gated group than in the true ECG-gated group (all *P* < 0.01) (Table 2).

**Table 2:** Comparison of examination time and radiation dose between true ECG-gated and virtual ECG-gated CCTA.

Groups	Positioning time (min)	Scan time (min)	Total time (min)	CTDIvol (mGy)	DLP (mGy.cm)	ED (mSv)
Virtual ECG-gated group (*n* = 126)	0.89 ± 0.26	2.02 ± 0.42	2.83 ± 0.37	18.95 ± 3.65	255.84 ± 49.31	3.58 ± 0.69
True ECG-gated group (*n* = 128)	2.03 ± 0.35	2.18 ± 0.56	4.15 ± 0.49	16.83 ± 2.87	227.21 ± 38.77	3.18 ± 0.54
*P*-value	<0.001	0.086	<0.001	<0.001	<0.001	<0.001

CTDIvol: Volume CT Dose Index. DLP: Dose Length Product. ED: Effective Dose.

### 3.3 Image quality

#### 3.3.1 Objective image quality

The measurements showed good interobserver agreement between the two readers, with all ICC values greater than 0.75. The virtual ECG-gated group had lower image noise than the true ECG-gated group; however, CT attenuation values of the aortic root and coronary artery segments did not differ significantly between the two groups (Table 3). The SNR and CNR of the aortic root and all coronary artery segments were significantly higher in the virtual ECG-gated group than in the true ECG-gated group (all *P* < 0.01) (Table 3).

**Table 3:** Comparison of objective image quality parameters between real ECG-gated and virtual ECG-gated CCTA.

Objective image quality parameters	Virtual ECG -gated group (*n* = 126)	True ECG –gated group (*n* = 128)	*P*-value
CT value (HU)			
AO	471.86 ± 63.74	464.82 ± 61.76	0.373
LM	440.05 ± 70.30	444.98 ± 58.16	0.544
LAD-M	420.09 ± 66.94	424.20 ± 56.09	0.597
LAD-D	389.25 ± 66.35	394.91 ± 49.04	0.441
LCX-M	414.11 ± 60.40	412.34 ± 57.69	0.811
LCX-D	366.27 ± 47.50	369.82 ± 51.35	0.568
RCA-P	435.23 ± 61.10	435.36 ± 54.68	0.986
RCA-M	412.19 ± 63.63	410.57 ± 50.85	0.823
RCA-D	370.89 ± 61.14	367.48 ± 50.04	0.627
Image noise	14.96 ± 3.34	19.47 ± 4.06	<0.001
SNR			
AO	32.88 ± 7.65	25.09 ± 7.07	<0.001
LM	30.77 ± 7.90	24.07 ± 6.94	<0.001
LAD-M	29.38 ± 7.67	22.95 ± 6.70	<0.001
LAD-D	27.16 ± 7.18	21.37 ± 6.08	<0.001
LCX-M	28.87 ± 6.93	22.33 ± 6.65	<0.001
LCX-D	25.62 ± 6.15	20.05 ± 6.11	<0.001
RCA-P	30.29 ± 7.04	23.50 ± 6.60	<0.001
RCA-M	28.78 ± 7.27	22.14 ± 6.10	<0.001
RCA-D	25.91 ± 6.77	19.85 ± 5.74	<0.001
CNR			
AO	28.94 ± 6.86	21.84 ± 6.39	<0.001
LM	26.82 ± 7.12	20.82 ± 6.26	<0.001
LAD-M	25.44 ± 6.89	19.71 ± 6.02	<0.001
LAD-D	23.22 ± 6.42	18.12 ± 5.40	<0.001
LCX-M	24.92 ± 6.16	19.08 ± 5.98	<0.001
LCX-D	21.68 ± 5.33	16.80 ± 5.43	<0.001
RCA-P	26.35 ± 6.26	20.25 ± 5.94	<0.001
RCA-M	24.83 ± 6.52	18.89 ± 5.45	<0.001
RCA-D	21.97 ± 6.00	16.60 ± 5.09	<0.001

#### 3.3.2 Subjective image quality

Good interobserver agreement was observed between the two readers. The kappa values for the subjective image quality scores of the LM, LAD, D1, LCX, OM1, RCA, PDA, PLB, and RI were 0.874, 0.821, 0.838, 0.863, 0.832, 0.833, 0.801, 0.895, and 0.857, respectively, all *P* < 0.05. There were no significant differences in subjective image quality scores for any coronary artery or major branch between the virtual ECG-gated and true ECG-gated groups (all *P* > 0.05), as shown in Table 4 and Figure 3.

**Table 4:** Comparison of subjective image quality parameters between real ECG-gated and virtual ECG-gated CCTA.

Coronary arteries	Virtual ECG -gated group (*n* = 126)	True ECG -gated group (*n* = 128)	*P*-value
LM	5 (5, 5)	5 (5, 5)	0.433
LAD	5 (5, 5)	5 (5, 5)	0.212
D1	5 (5, 5)	5 (5, 5)	0.841
LCX	5 (5, 5)	5 (5, 5)	0.115
OM1	5 (5, 5)	5 (4, 5)	0.827
RCA	5 (5, 5)	5 (5, 5)	0.273
PDA	5 (4, 5)	5 (4, 5)	0.772
PLB	5 (4, 5)	5 (4, 5)	0.361
RI	5 (5, 5)	5 (5, 5)	0.354

**Figure 3:**
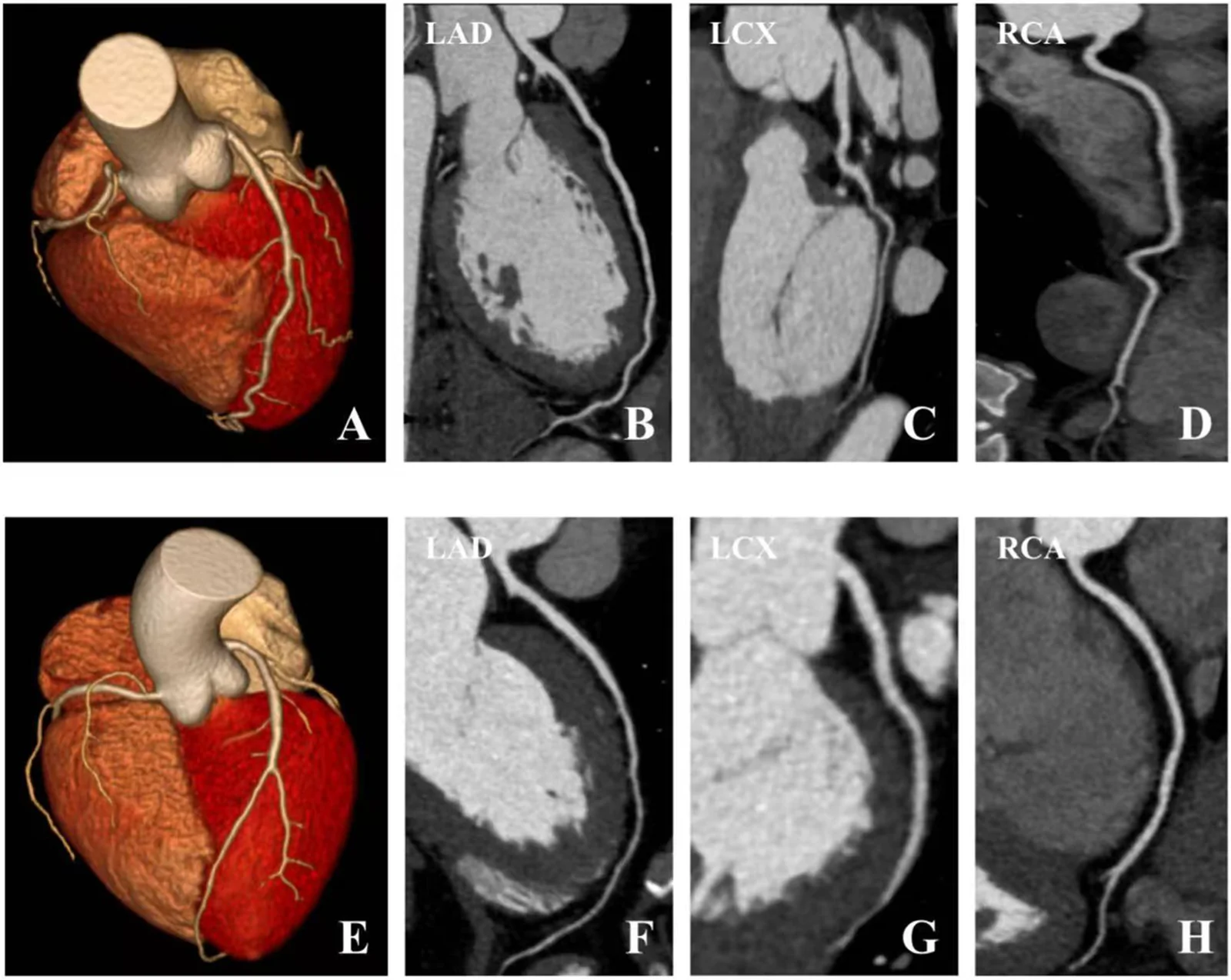
**(A–D)** show CCTA images acquired with true ECG gating in a 55-year-old male patient with a height of 1.70 m, body weight of 76 kg, BMI of 26.30 kg/m², mean heart rate of 69 bpm, scan length of 14 cm, and effective dose of 3.15 mSv. **(E–H)** show CCTA images acquired with virtual ECG gating in a 56-year-old male patient with a height of 1.68 m, body weight of 76 kg, BMI of 26.93 kg/m², mean heart rate of 70 bpm, scan length of 14 cm, and effective dose of 3.44 mSv.

### 3.4 Diagnostic performance

Among the enrolled patients, 51 patients underwent ICA within 30 days after CCTA. Four major coronary arteries were evaluated for each patient, resulting in 100 coronary vessels in the virtual ECG-gated group (25 patients) and 104 coronary vessels in the true ECG-gated group (26 patients). Because coronary arteries within the same patient were considered correlated observations, diagnostic performance was analyzed using patient-level cluster bootstrap resampling and mixed-effects logistic regression models. In the virtual ECG-gated group, 88 of 100 coronary vessels were correctly classified, yielding a diagnostic accuracy of 88.00%. In the true ECG-gated group, 83 of 104 coronary vessels were correctly classified, yielding a diagnostic accuracy of 79.81%. The diagnostic performance metrics are summarized in Table 5. The AUC were 0.89 (95% CI: 0.82–0.96) and 0.82 (95% CI: 0.74–0.91) in virtual and true ECG-gated group, respectively. The ROC curves demonstrated comparable diagnostic performance between virtual ECG-gated and true ECG-gated CCTA (Figure 4). Figure 5 presents a representative case showing the comparison between invasive coronary angiography and virtual ECG-gated CCTA images.

**Table 5:** Diagnostic performance was analyzed on a vessel level.

Groups	AUC （95% CI）	Sensitivity （95% CI）	Specificity （95% CI）	PPV （95% CI）	NPV （95% CI）	Accuracy （95% CI）
Virtual ECG -gated	0.89 (0.82, 0.96)	0.87 (0.77, 0.98)	0.91 (0.82, 0.99)	0.97 (0.94, 0.99)	0.678 (0.61, 0.82)	0.88 (0.81, 0.98)
True ECG -gated	0.82 (0.74, 0.91)	0.77 (0.65, 0.89)	0.88 (0.77, 0.97)	0.95 (0.91, 0.98)	0.55 (0.44, 0.76)	0.80 (0.71, 0.89)

**Figure 4:**
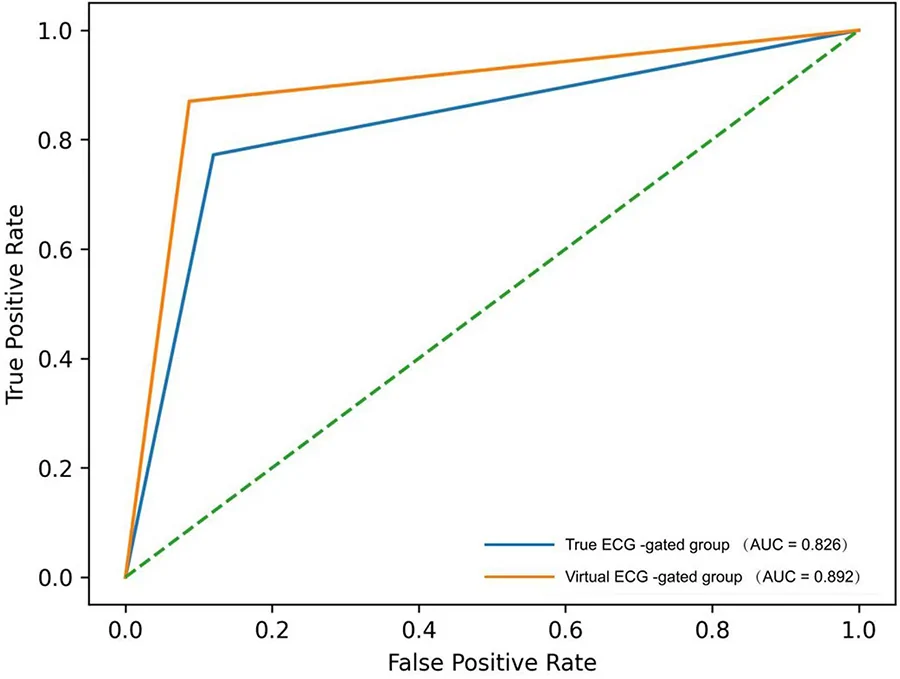
ROC curves of true ECG-gated and virtual ECG-gated CCTA for diagnostic performance.

**Figure 5:**
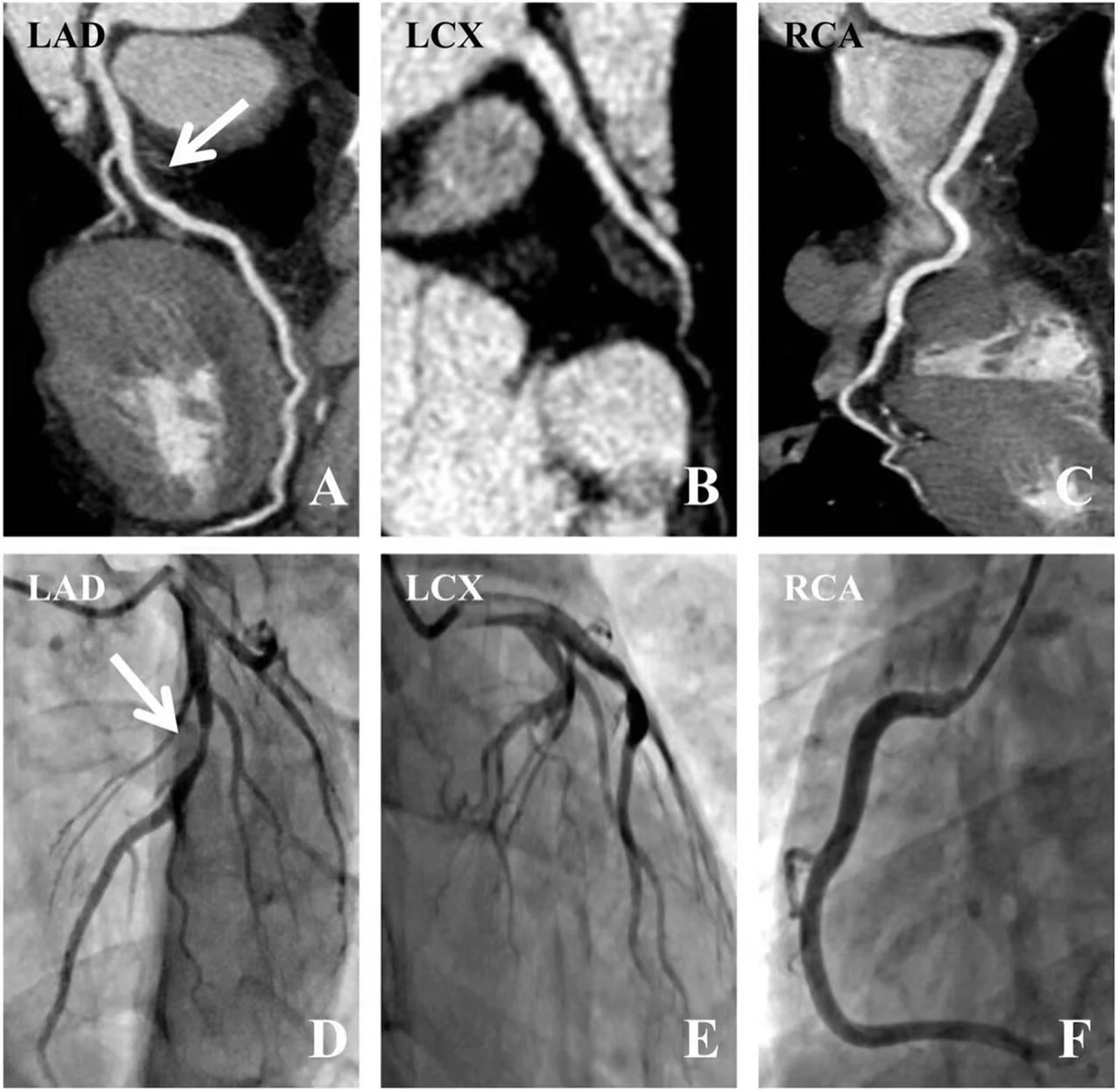
Comparison between virtual ECG-gated CCTA and ICA. A 43-year-old male patient with a mean heart rate of approximately 78 bpm. Virtual ECG-gated CCTA images **(A–C)** showed findings consistent with ICA images **(D–F)**. Images A and D show the pLAD, where a non-calcified plaque is visible (white arrow). CCTA showed approximately 70% luminal stenosis, while ICA demonstrated 70% -80% stenosis. Images B and E show the LCX, and images C and F show the RCA, with no obvious plaque or stenosis observed.

## Discussion

This study demonstrated that virtual ECG-gated CCTA effectively shortened examination time while maintaining diagnostic performance. Compared with true ECG-gated CCTA, virtual ECG-gated CCTA was associated with a slightly higher radiation dose; however, the optimized virtual ECG-gated protocol still achieved comparable objective image quality, with favorable quantitative parameters including lower background image noise and higher SNR and CNR. Subjective image quality scores and diagnostic agreement showed no significant differences between the two groups. These findings suggest that optimized virtual ECG-gated CCTA can achieve a favorable balance between examination efficiency and diagnostic image quality.

Virtual ECG-gated CCTA generates an artificial simulated ECG signal based on the pulse or heart rate measured before the examination, without the need for attaching surface ECG electrodes. This simulated ECG signal is then used for “virtual gating” reconstruction of data acquired over a complete cardiac cycle. Unlike conventional true ECG-gated CCTA, which relies on real-time ECG signal acquisition, virtual ECG-gated CCTA avoids several procedural steps, including electrode attachment, ECG signal adjustment, and waiting for signal stabilization. Therefore, the workflow is simplified, resulting in a marked reduction in preparation time and overall examination time (15, 22). This technical advantage may be particularly important in emergency settings or clinical scenarios requiring a highly efficient workflow.

The simulated ECG signal was generated based on the heart rate estimated before CCTA acquisition, transient heart rate variations during breath-holding, contrast injection, or patient anxiety may occur during scanning. However, the simulated ECG signal in ECG-less CCTA is primarily used as a temporal reference for virtual gating rather than a direct substitute for real-time ECG monitoring. By using an acquisition window covering the entire cardiac cycle, sufficient cardiac data can be obtained despite minor heart rate fluctuations. Furthermore, automatic phase selection by Smart Phase and coronary motion correction using SSF further compensate for residual phase mismatch and motion artifacts, contributing to the robustness of ECG-less CCTA reconstruction. Although the simulated ECG signal does not represent the patient's real-time electrical cardiac activity, it provides a reliable temporal framework for cardiac phase identification and motion compensation. Previous studies have demonstrated the feasibility of this approach for coronary CT angiography, supporting its clinical applicability (22). With the development of wide-detector CT systems, high-speed gantry rotation, automatic optimal phase selection, and coronary motion correction algorithms, virtual ECG-gated CCTA now has a strong technical foundation. These advances provide support for acquiring high-quality coronary artery images even in the absence of real-time ECG signals (23, 24). Therefore, from the perspectives of both technical principle and scanner capability, virtual ECG-gated CCTA has good technical feasibility.

In the present study, the NI was set at 18 in the virtual ECG-gated CCTA group and 20 in the true ECG-gated CCTA group. NI is a dimensionless parameter closely related to CTDI and image quality. Based on the scout image, reference patient size, and the patient's actual body habitus, NI is used by the automatic tube current modulation system to adjust the tube current–time product (25). Reducing the NI decreases image noise and improves image quality; however, this improvement is achieved at the cost of increased radiation exposure (26). This finding suggests that reducing the NI may effectively decrease background tissue interference, thereby improving the contrast between the coronary lumen and surrounding tissues. Accordingly, the optimized virtual ECG-gated CCTA protocol achieved comparable objective image quality to true ECG-gated CCTA. The favorable quantitative parameters observed in the virtual ECG-gated group may reflect the combined effects of the gating strategy and protocol optimization. This finding is consistent with the comparable subjective image quality scores and diagnostic agreement rates observed between the virtual ECG-gated and true ECG-gated groups.

Furthermore, virtual ECG-gated CCTA may offer particular advantages in patients with arrhythmias (e.g., atrial fibrillation or frequent ectopic beats) or contraindications to *β*-blockers (e.g., asthma, severe chronic obstructive pulmonary disease, or hypotension). Conventional ECG-gated CCTA remains challenging in these populations due to irregular cardiac rhythms or difficulties in achieving adequate heart rate control, and some patients may require direct referral for ICA. Virtual ECG-gated CCTA eliminates the need for real-time ECG signal acquisition and *β*-blocker administration for heart rate optimization, thereby simplifying patient preparation, reducing examination workflow time, and expanding the applicability of CCTA in these clinically challenging populations, particularly in elderly patients or those with multiple comorbidities who cannot tolerate heart rate-lowering medications.

The higher radiation dose observed in the virtual ECG-gated group may be mainly related to the use of a relatively wide acquisition window. Although virtual ECG-gated CCTA was associated with a higher effective radiation dose than true ECG-gated CCTA, the increase was relatively modest, with only an approximately 13% increase observed in our study. Furthermore, compared with previously reported radiation doses, the effective dose of virtual ECG-gated CCTA in the present study remained within the range of modern low-dose CCTA protocols and was comparable to or even lower than that reported for some third-generation dual-source CT systems (27). Wang et al. reported that narrowing the acquisition window from 200 to 750 ms to 300–600 ms reduced the effective dose by 28.3%, and the ED was no longer significantly different from that of true ECG-gated CCTA (*P* = 0.62) (15). However, in emergency patients with acute chest pain, true ECG-gated CCTA may not always be feasible. In some critically ill patients, reliable ECG signal acquisition can be difficult because of unstable ECG signals, challenges in attaching surface electrodes, or interference from emergency life-support measures (28).

Virtual ECG-gated CCTA can effectively address this issue by eliminating the need for real-time ECG signal acquisition, while also shortening examination time and improving workflow efficiency. Although the observed reduction in examination time may appear limited in routine clinical practice, its clinical relevance may be more pronounced in emergency settings, where rapid imaging assessment is essential for early diagnosis, patient triage, and treatment planning. By reducing preparation-related procedures, virtual ECG-gated CCTA may provide additional time for clinical decision-making, particularly in patients with acute chest pain requiring expedited evaluation. Therefore, although it is associated with a slightly higher radiation dose, this trade-off may be clinically reasonable in selected clinical scenarios. These findings suggest that, in future clinical applications, virtual ECG-gated CCTA should be combined with radiation dose-reduction strategies, such as individualized narrowing of the acquisition window, automatic tube current modulation, low-tube-voltage protocols, and deep learning-based reconstruction algorithms. Such optimization may help minimize radiation exposure while maintaining image quality, thereby achieving an optimal balance between diagnostic image quality and radiation safety.

This study has several limitations. First, the sample size was relatively small and all patients were enrolled from a single center, which may have introduced potential selection bias. Future studies with larger sample sizes and multicenter datasets are warranted to further validate our findings. Second, although alternating date-based allocation reduced potential operator preference during enrollment, it was not equivalent to true randomization and may have introduced residual selection bias related to temporal factors, including workflow characteristics, staffing patterns, scanner availability, and referral pathways. Third, additional radiation dose-reduction techniques were not incorporated in the present study. Future research should further combine virtual ECG-gated CCTA with artificial intelligence-based techniques, such as deep learning reconstruction and intelligent motion correction, to explore optimized scanning protocols that can improve image quality while reducing radiation dose (29). Furthermore, because ICA was performed only in clinically indicated patients rather than in the entire cohort, potential selection and verification bias cannot be excluded, which may limit the generalizability of the diagnostic performance findings.

## 4 Conclusion

Virtual ECG-gated CCTA based on a wide-detector CT platform demonstrated promising clinical applicability by reducing examination workflow time while preserving diagnostic performance. With an optimized acquisition protocol, virtual ECG-gated CCTA provided adequate objective and subjective image quality for coronary evaluation. The potential improvement in objective image quality should be interpreted cautiously, as it may reflect the combined effects of virtual ECG-gating and protocol optimization rather than virtual ECG-gating alone. Although virtual ECG-gated CCTA resulted in a slightly higher radiation dose, its ability to shorten scanning time may offer clinical value for patients with acute chest pain, particularly in emergency settings where timely diagnosis and management are critical.

## Data Availability

The raw data supporting the conclusions of this article will be made available by the authors, without undue reservation.
